# Supplementary material for: Striatal Dopamine at Learned Sequence Boundaries Sustains Birdsong
Source: bioRxiv. 2026 Jul 5:2026.07.05.736606. Preprint. [Version 1] doi: 10.64898/2026.07.05.736606 (PMC13345225; doi:10.64898/2026.07.05.736606)
Supplement: Supplement 1 [file NIHPP2026.07.05.736606v1-supplement-1.pdf]

bioRxiv preprint doi: https://doi.org/10.64898/2026.07.05.736606; this version posted July 6, 2026. The copyright holder for this preprint (which was not certified by peer review) is the author/funder, who has granted bioRxiv a license to display the preprint in perpetuity. It is made available under a CC-BY 4.0 International license.

Supplementary Figures

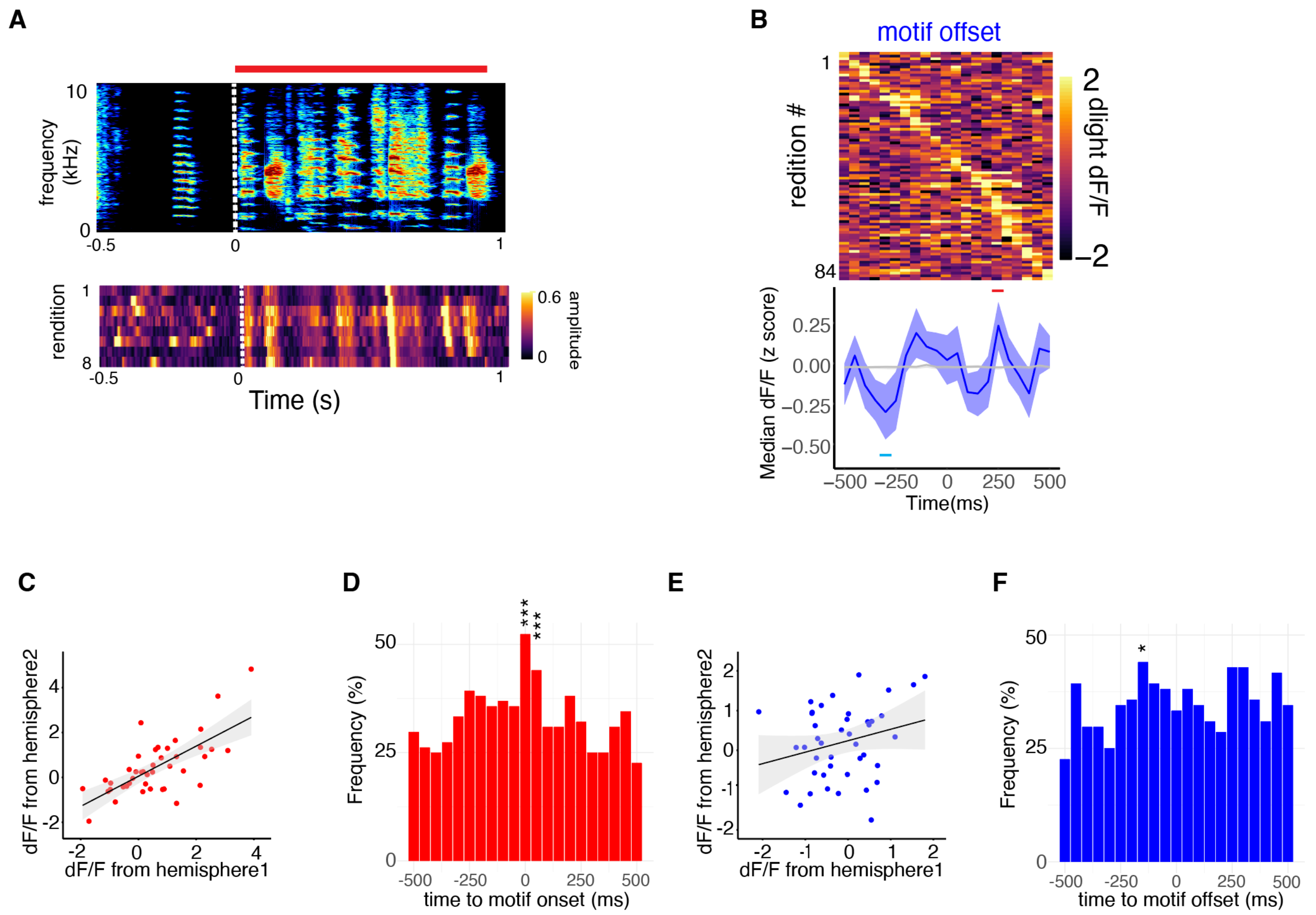

Figure S1 Motif-Aligned Dopamine Dynamics During Directed Singing.

- **A)** Top, example spectrogram of a song motif aligned to motif onset. The red bar marks the motif, and the white dashed line indicates motif onset. Bottom, sound-amplitude stack plot of eight motif renditions aligned to motif onset, illustrating the temporal consistency of motif structure across renditions.
- **B)** Trial-by-trial dLight fluorescence aligned to motif offset during directed singing and sorted by peak timing (n = 84 renditions). Bottom, median motif-offset-aligned dLight activity. At motif offset, shuffle analysis detected a significant post-offset peak at 250 ms (red bar; $p = 0.010$; other time points, $p > 0.05$) and a significant pre-offset dip at –300 ms (blue bar; $p = 0.014$). Shaded error indicates ±s.e.m.; grey shading indicates the 95% shuffle CI.
- **C)** Across-hemisphere correlation of motif-onset-aligned dLight fluorescence. Dopamine signals were significantly correlated between hemispheres near motif onset ($R = 0.60$, $p = 3.7 \times 10^{-5}$).
- **D)** Frequency histogram of detected dLight transients aligned to motif onset. Transients occurred significantly more often near motif onset, with enrichment at 0 ms ($p < 0.001$) and 50 ms ($p = 0.006$; shuffle test).
- **E)** Across-hemisphere correlation of motif-offset-aligned dLight fluorescence. Correlation between hemispheres at motif offset was weak and not significant ($R = 0.21$, $p = 0.19$).
- **F)** Frequency histogram of detected dLight transients aligned to motif offset. No consistent increase in transient frequency was observed around motif offset, although a weak enrichment occurred before offset at –150 ms ($p = 0.022$; shuffle test).

bioRxiv preprint doi: https://doi.org/10.64898/2026.07.05.736606; this version posted July 6, 2026. The copyright holder for this preprint (which was not certified by peer review) is the author/funder, who has granted bioRxiv a license to display the preprint in perpetuity. It is made available under a CC-BY 4.0 International license.

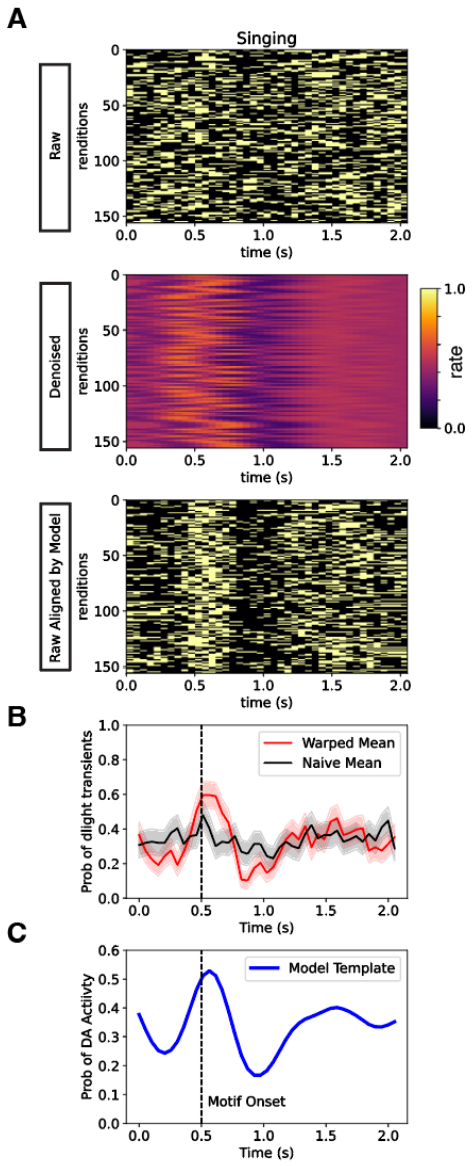

Figure S2. Motif-Aligned Dopamine Transients During Singing.

- **A)** Trial-by-trial binary dopamine-transient events during singing, shown as raw event rasters (top), denoised transient-rate representations (middle), and raw event rasters realigned by the shift-only time-warping model (bottom). Each row represents one motif rendition.
- **B)** Mean probability of dopamine transients across motif renditions using naive alignment (black) or shift-only model alignment (red). Shaded areas indicate 95% CI. Dashed vertical line marks motif onset.
- **C)** Model-derived transient-probability template during singing. Dopamine transients showed a consolidated peak near motif onset followed by a dip within the motif. Dashed vertical line marks motif onset.

bioRxiv preprint doi: https://doi.org/10.64898/2026.07.05.736606; this version posted July 6, 2026. The copyright holder for this preprint (which was not certified by peer review) is the author/funder, who has granted bioRxiv a license to display the preprint in perpetuity. It is made available under a CC-BY 4.0 International license.

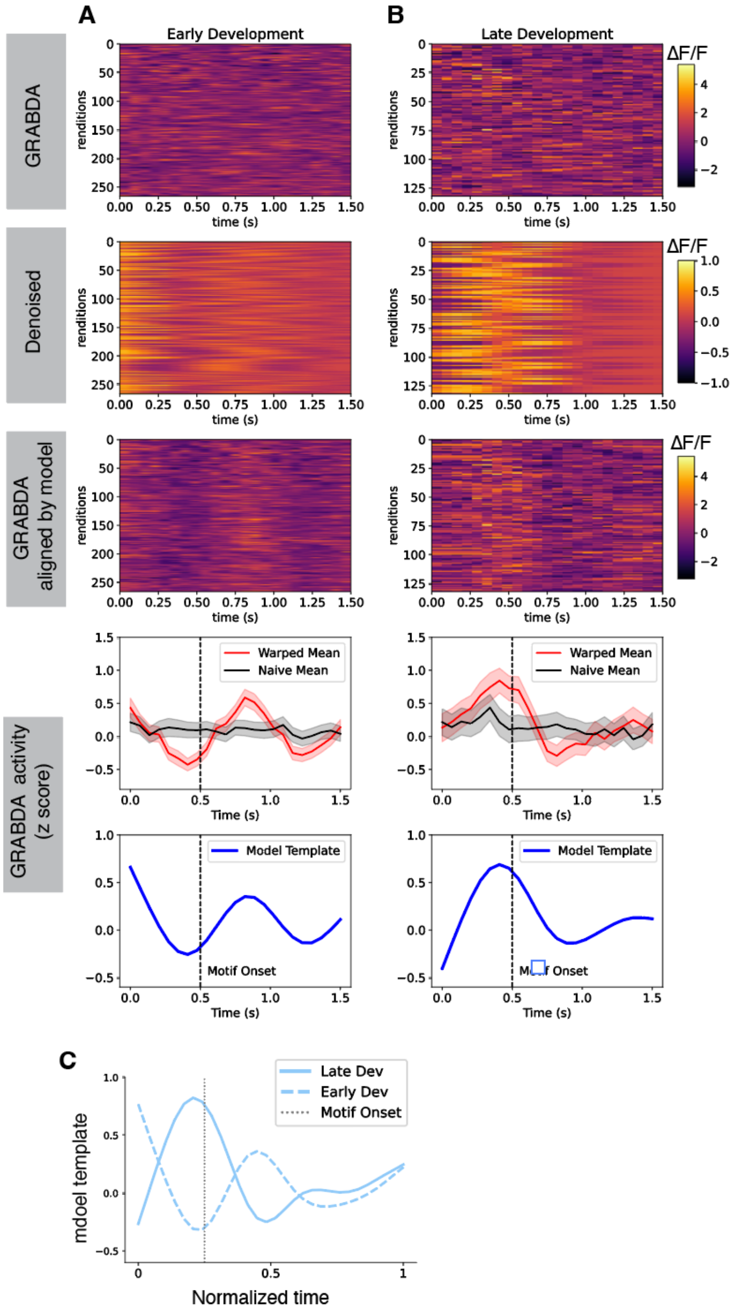

Figure S3. Alignment of GRAB-DA Signals During Song Development.

- **A)** Trial-by-trial GRAB-DA fluorescence from early-development song, shown as raw fluorescence traces (top), denoised representations (second row), raw traces realigned by the shift-only time-warping model (third row), naive versus model-aligned mean activity (fourth row), and the model-derived activity template (bottom). Each row represents one motif rendition. Dashed vertical line marks motif onset.
- **B)** Trial-by-trial GRAB-DA fluorescence from late-development song, shown as raw fluorescence traces, denoised representations, model-aligned raw traces, naive versus model-aligned mean activity, and the model-derived activity template as in (**A**). Late-development song showed a consolidated dopamine peak near motif onset.
- **C)** Model-derived GRAB-DA templates plotted in normalized motif time for early- and late-development song. The dopamine activity peak was offset-biased in early development and shifted toward motif onset by late development.

bioRxiv preprint doi: https://doi.org/10.64898/2026.07.05.736606; this version posted July 6, 2026. The copyright holder for this preprint (which was not certified by peer review) is the author/funder, who has granted bioRxiv a license to display the preprint in perpetuity. It is made available under a CC-BY 4.0 International license.

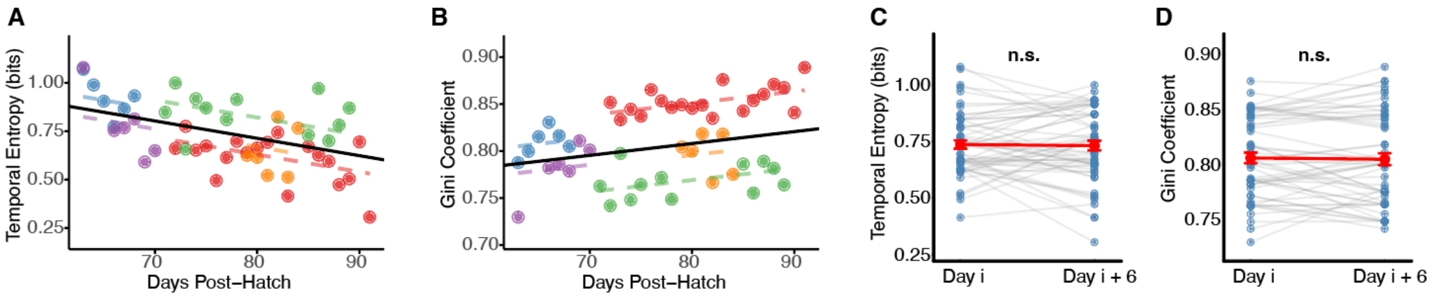

Figure S4. Developmental Changes in Temporal Concentration of Dopamine Transients.

A) – **B**) Population-level analysis of dopamine-transient temporal concentration across vocal development. Linear mixed-effects models showed that temporal entropy decreased with age (**A**), whereas the Gini coefficient increased with age (**B**) (n = 49 observations from 5 birds, 63 to 91 dph). Each colored dot represents the mean value for one bird at a given age; colors indicate individual birds. Black lines show population-level fixed effects from LMMs with bird identity as a random intercept.

C) – **D**) Paired comparison of temporal concentration metrics between the first day (Day i) and last day (Day i + 6) of 6-day sliding windows. Gray lines connect paired observations from individual birds within the same temporal window (n = 57 paired observations from 5 birds across 24 windows). Red symbols indicate group means ± SEM. Temporal entropy (**C**) and Gini coefficient (**D**) did not change significantly within 6-day windows.
Statistics are summarized in Table 1.

bioRxiv preprint doi: https://doi.org/10.64898/2026.07.05.736606; this version posted July 6, 2026. The copyright holder for this preprint (which was not certified by peer review) is the author/funder, who has granted bioRxiv a license to display the preprint in perpetuity. It is made available under a CC-BY 4.0 International license.

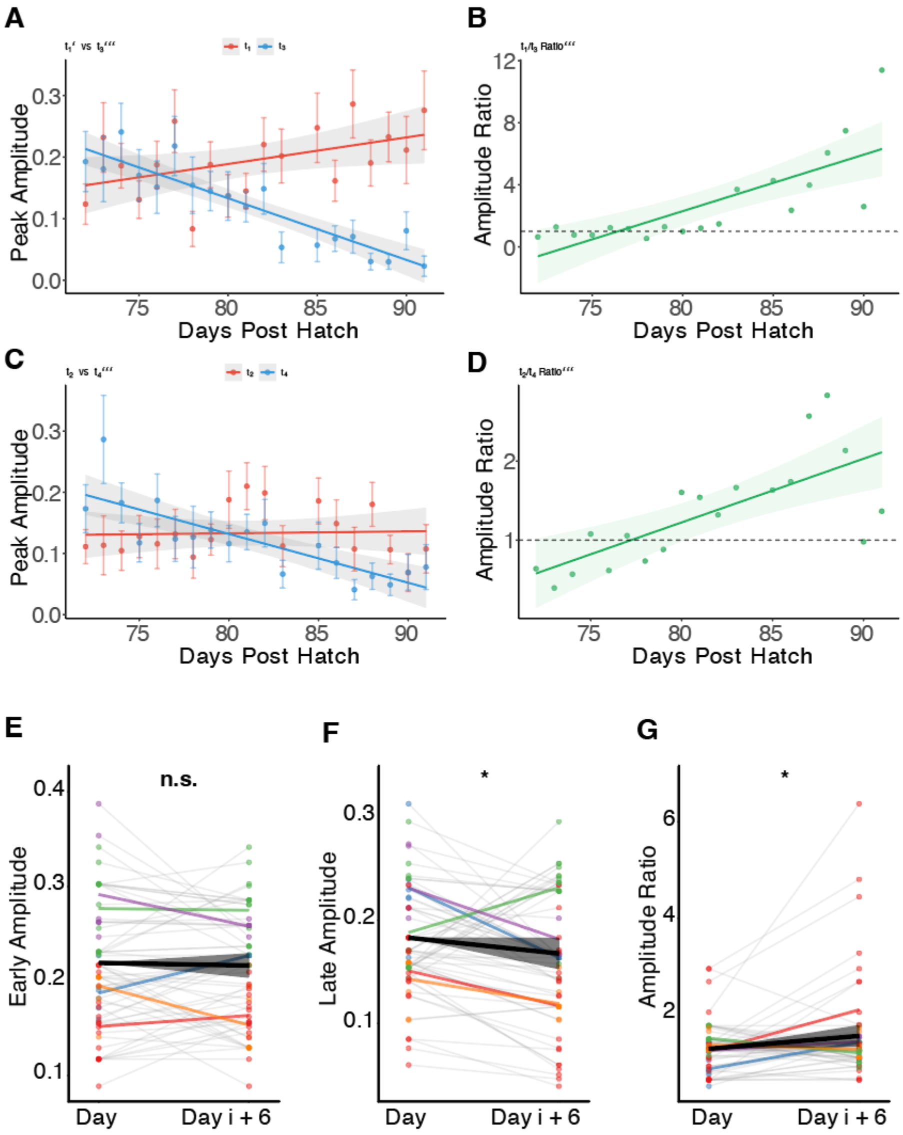

Figure S5 Dopamine Amplitude Shifts Across Motif Segments During Vocal Development

A) – **D**) Representative example from a single bird showing amplitude redistribution across different pairs of motif segment. (**A**) Peak amplitudes for early (t_1, red) and late (t_3, blue) segments across development. Early amplitude increased significantly while late amplitude decreased significantly. (**B**) Early/late amplitude ratio (t_1/t_3) increased significantly across development. (**C**) Peak amplitudes for early (t_2, red) and late (t_4, blue) segments showing developmental trajectory. Early amplitude showed no significant change while late amplitude decreased significantly. (**D**) Early/late amplitude ratio (t_2/t_4) increased significantly across development.

E) – **G**) Population-level within-window paired analysis using mixed-effects models across 6-day sliding windows. Points: individual observations colored by animal. Gray lines: individual observations within each window (animal × window combinations). Colored lines: mean trajectory for each animal across all windows. Black line with ribbon: population-level trend with 95% CI from fixed effects. (**E**) Early amplitude showed no significant within-window change. (**F**) Late amplitude decreased significantly within windows. (**G**) Amplitude ratio increased significantly within windows.

Statistics are summarized in Table 1.

bioRxiv preprint doi: https://doi.org/10.64898/2026.07.05.736606; this version posted July 6, 2026. The copyright holder for this preprint (which was not certified by peer review) is the author/funder, who has granted bioRxiv a license to display the preprint in perpetuity. It is made available under a CC-BY 4.0 International license.

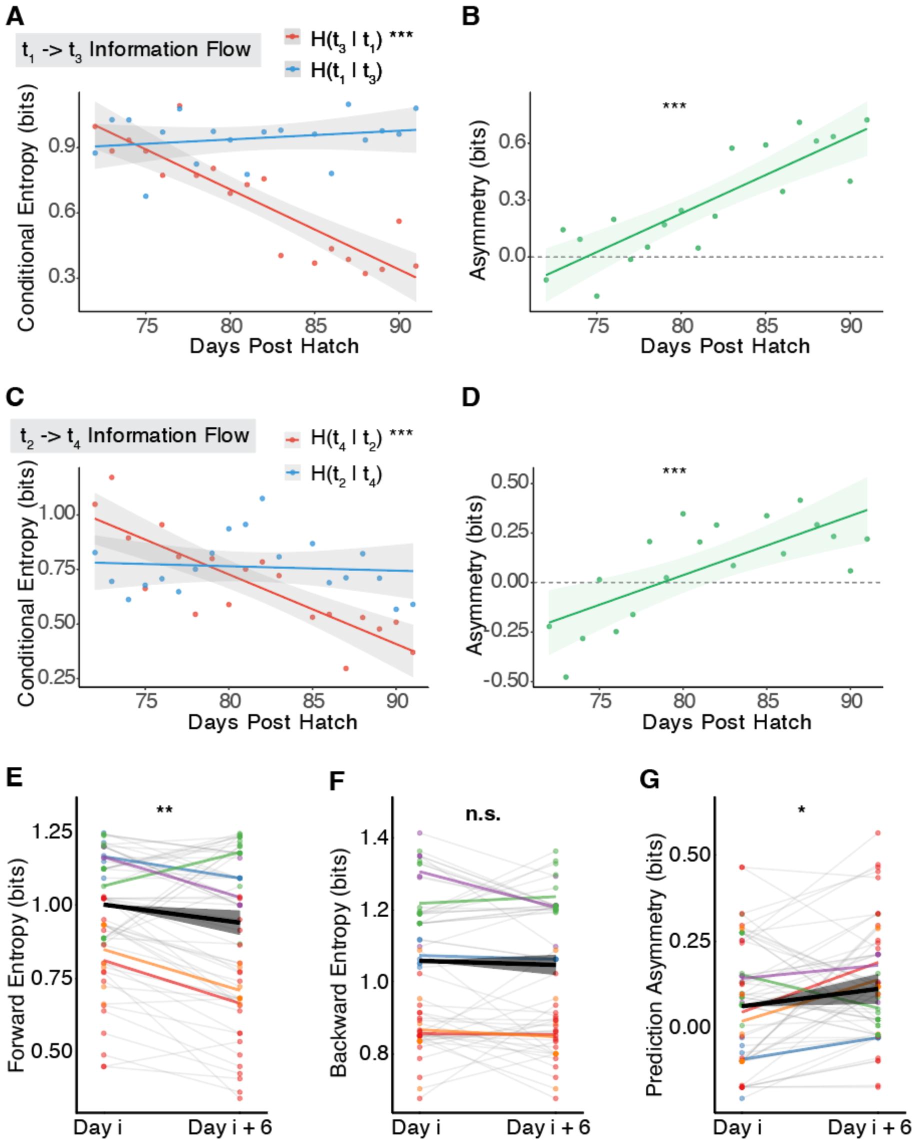

Figure S6 Directional Information Flow Pattern across Different Motif Segments During Vocal Development

A) – **D**) Representative example from a single bird showing directional information flow between different pairs of motif segments. (**A**) Conditional entropies for forward ($H(t_3|t_1)$, red) and backward ($H(t_1|t_3)$, blue) prediction across development. Forward entropy decreased significantly, indicating improved predictability of t_3 from t_1, while backward entropy showed no significant change. (**B**) Prediction asymmetry ($H(t_1|t_3) - H(t_3|t_1)$) increased significantly across development, with dominance crossover at DPH 74.4. (**C**) Conditional entropies for forward ($H(t_4|t_2)$, red) and backward ($H(t_2|t_4)$, blue) prediction showing developmental trajectory. Forward entropy decreased significantly, while backward entropy showed no significant change. (**D**) Prediction asymmetry ($H(t_2|t_4)$ - $H(t_4|t_2)$) increased significantly across development, with dominance crossover at DPH 78.7.

E) – **G**) Population-level within-window paired analysis using mixed-effects models across 6-day sliding windows. Points: individual observations colored by animal. Gray lines: individual observations within each window (animal × window combinations). Colored lines: mean trajectory for each animal across all windows. Black line with ribbon: population-level trend with 95% CI from fixed effects. (**E**) Forward entropy decreased significantly within windows, indicating improved forward predictability. (**F**) Backward entropy showed no significant within-window change. (**G**) Prediction asymmetry increased significantly within windows, demonstrating a shift toward forward prediction dominance.

Statistics are summarized in Table 1.

bioRxiv preprint doi: https://doi.org/10.64898/2026.07.05.736606; this version posted July 6, 2026. The copyright holder for this preprint (which was not certified by peer review) is the author/funder, who has granted bioRxiv a license to display the preprint in perpetuity. It is made available under a CC-BY 4.0 International license.

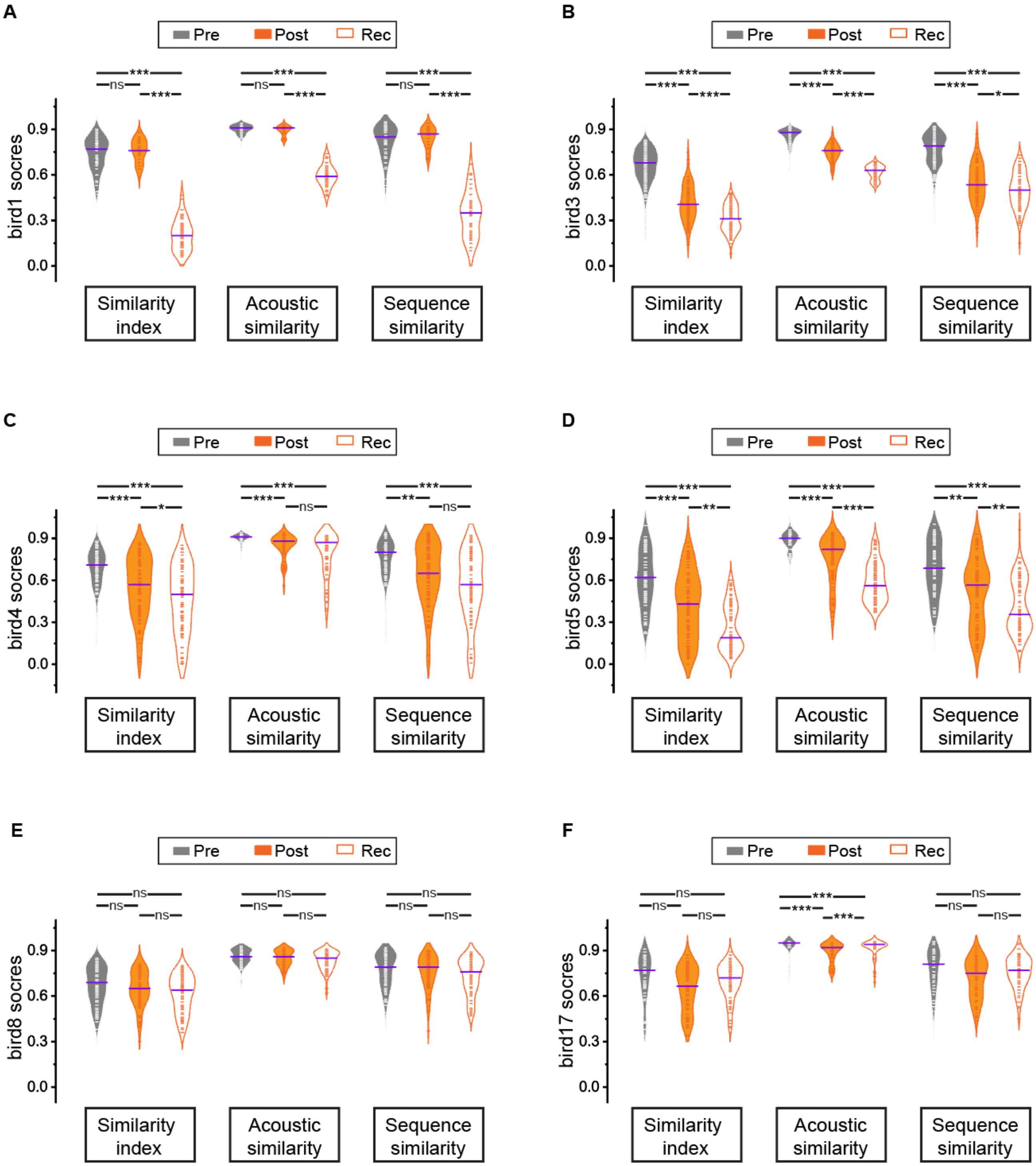

Figure S7 Individual Variability in Similarity Metrics across Pre, Post, and Rec Periods in ArchT+ Birds.

A) – **F**) Violin plots of similarity index, acoustic similarity, and sequence similarity scores for individual ArchT+ birds (Bird ID: 1, 3, 4, 5, 8, 17) recorded at Pre, Post, and Rec periods. Lines indicate median scores. Statistical comparisons for each bird are shown in Table 2(Mood's tests).

bioRxiv preprint doi: https://doi.org/10.64898/2026.07.05.736606; this version posted July 6, 2026. The copyright holder for this preprint (which was not certified by peer review) is the author/funder, who has granted bioRxiv a license to display the preprint in perpetuity. It is made available under a CC-BY 4.0 International license.

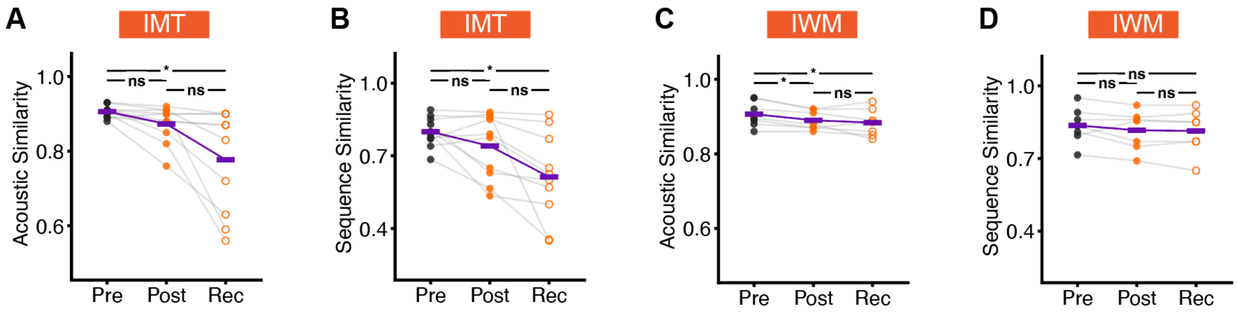

Figure S8. Acoustic and Sequence Similarity After Dopamine Inhibition at Motif Transitions or Within Motifs.

A) – **B**). Acoustic similarity (**A**) and sequence similarity (**B**) across Pre, Post, and Rec periods in IMT birds. IMT birds showed progressive reductions in both acoustic and sequence similarity after optogenetic inhibition.

C) – **D**). Acoustic similarity (**C**) and sequence similarity (**D**) across Pre, Post, and Rec periods in IWM birds. IWM birds showed limited disruption, with no progressive decline in sequence similarity. Gray lines indicate individual birds; purple lines indicate group means.

Statistics are summarized in Table 3.

bioRxiv preprint doi: https://doi.org/10.64898/2026.07.05.736606; this version posted July 6, 2026. The copyright holder for this preprint (which was not certified by peer review) is the author/funder, who has granted bioRxiv a license to display the preprint in perpetuity. It is made available under a CC-BY 4.0 International license.

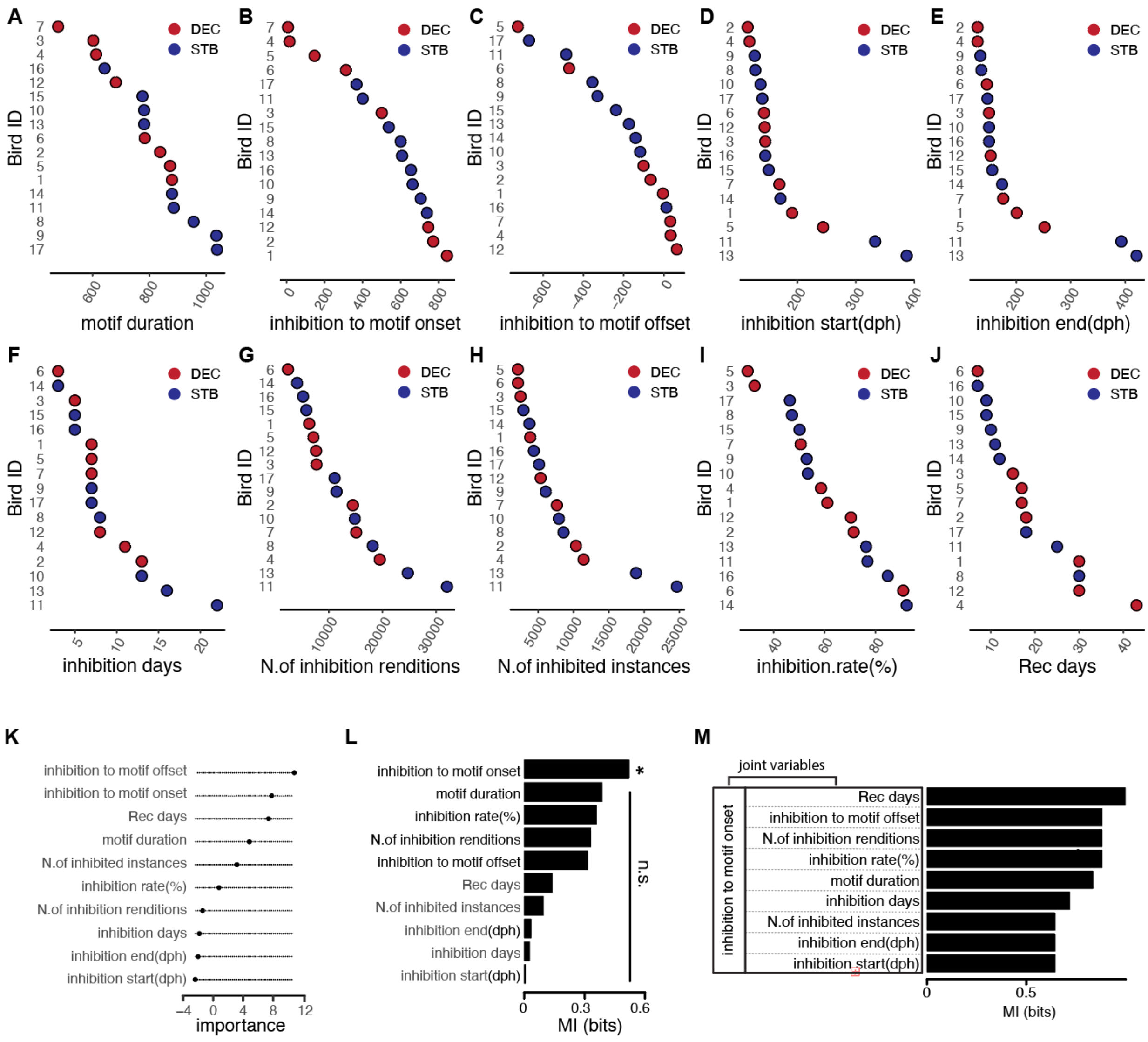

Figure S9. Experimental Variables Associated with Song Decrystallization after Dopamine Inhibition.

- **A)** – **J**) ArchT+ birds sorted by experimental and behavioral variables and labeled by endpoint behavioral outcome. Red points indicate decrystallized (**DEC**) birds; blue points indicate stable (**STB**) birds. Variables include motif duration (**A**), inhibition timing relative to motif onset (**B**), inhibition timing relative to motif offset (**C**), inhibition start age (**D**), inhibition end age (**E**), number of inhibition days (**F**), number of inhibited song renditions (**G**), number of inhibited instances (**H**), inhibition rate (**I**), and recovery duration (**J**).
- **K)** Random forest feature importance for classifying **DEC** versus **STB** birds. Inhibition timing relative to motif offset and onset, and recovery duration ranked among the strongest predictors. Points indicate feature-importance estimates.
- **L)** Mutual information (**MI**) between individual experimental variables and **DEC/STB** outcome. Inhibition timing relative to motif onset was the only variable significantly associated with behavioral outcome ($p = 0.032$; all other variables, $p > 0.05$).
- **M)** Joint-variable **MI** analysis using inhibition timing relative to motif onset paired with each additional variable. The largest MI was observed for motif-onset timing combined with recovery duration, consistent with progressive decrystallization after perturbation near motif onset.

bioRxiv preprint doi: https://doi.org/10.64898/2026.07.05.736606; this version posted July 6, 2026. The copyright holder for this preprint (which was not certified by peer review) is the author/funder, who has granted bioRxiv a license to display the preprint in perpetuity. It is made available under a CC-BY 4.0 International license.

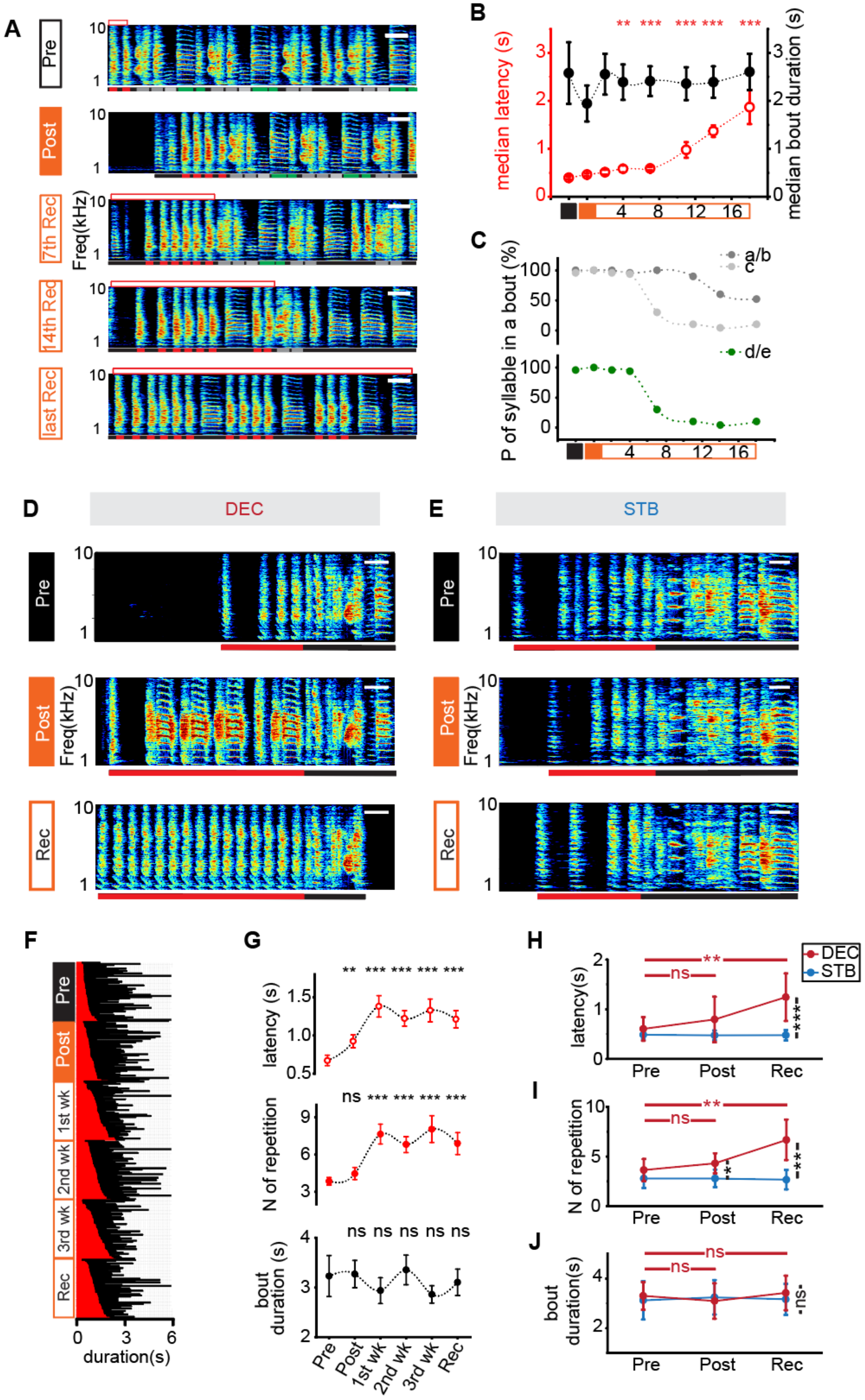

Figure S10. Progressive Song Changes Following Phasic Inhibition of Dopamine Signaling at Motif Transitions.

A) Representative spectrograms from a **DEC** bird across Pre, Post, 7th Rec, 14th Rec, and last Rec sessions. Red boxes above each spectrogram indicate the latency to motif initiation; Red bars mark introductory notes; green bars mark syllables that were progressively lost; gray bars mark retained song elements. Scale bar, 100 ms.

bioRxiv preprint doi: https://doi.org/10.64898/2026.07.05.736606; this version posted July 6, 2026. The copyright holder for this preprint (which was not certified by peer review) is the author/funder, who has granted bioRxiv a license to display the preprint in perpetuity. It is made available under a CC-BY 4.0 International license.

B) Quantification for the **DEC** bird shown in (**A**). Motif-initiation latency increased progressively during recovery, whereas bout duration remained stable. Red, latency to motif initiation; black, bout duration.

C) Probability of syllable occurrence for the **DEC** bird shown in (**A**). Specific learned syllables were progressively omitted during recovery.

D) Example spectrograms from a **DEC** bird across Pre, Post, and Rec periods. Red bars mark introductory notes; black bars mark the first motif. Scale bar, 100 ms.

E) Example spectrograms from a **STB** bird across Pre, Post, and Rec periods. Red and black bars mark introductory notes and the first motif, respectively. Scale bar, 100 ms.

F) Example song bouts from the **DEC** bird shown in (**D**), illustrating progressive prolongation of motif-initiation latency across recovery. Red marks latency to motif initiation; black marks bout duration.

G) Quantification for the **DEC** bird shown in (**D**). Motif-initiation latency and introductory-note repetitions increased after inhibition and during recovery, whereas bout duration remained unchanged.

H) – **J**). Group analyses of motif-initiation latency (**H**), number of introductory-note repetitions before the first motif (**I**), and bout duration (**J**) across Pre, Post, and Rec periods in **DEC** and **STB** birds. **DEC** birds showed progressive increases in latency and introductory-note repetitions, whereas bout duration remained unchanged. **STB** birds showed no comparable changes. Data are mean ± SEM.

Statistics are summarized in Table 4.

bioRxiv preprint doi: https://doi.org/10.64898/2026.07.05.736606; this version posted July 6, 2026. The copyright holder for this preprint (which was not certified by peer review) is the author/funder, who has granted bioRxiv a license to display the preprint in perpetuity. It is made available under a CC-BY 4.0 International license.

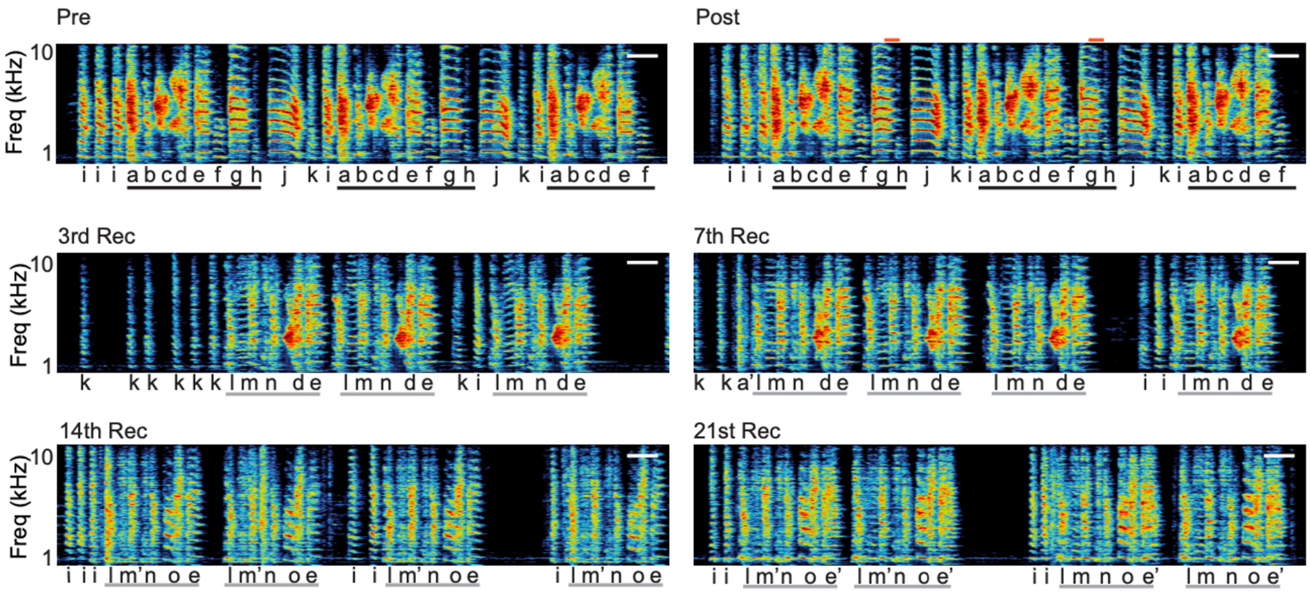

Figure S11. Progressive Loss of Learned Song Structure After Motif-Transition Dopamine Inhibition.

Expanded example from the **DEC** bird shown in Fig. **3C**, with additional recovery sessions and syllable annotations. Spectrograms show progressive omission or replacement of learned vocal components from Pre and Post through 3rd, 7th, 14th, and 21st Rec sessions. Individual syllables are annotated alphabetically; i marks introductory notes, and syllable variants are indicated by primes (a’, e’, m’). Black lines denote baseline motifs, and gray lines indicate altered motifs. Scale bars, 100 ms.
